# Bonding performance of glass ionomer cement to carious dentin treated with different surface treatment protocols using silver diamine fluoride

**DOI:** 10.1038/s41598-023-41511-9

**Published:** 2023-08-30

**Authors:** Jarinya Rinsathon, Suthinee Wiriyasuebpong, Kunwara Thariya, Panupong Jiradechochai, Porada Phetsuk, Sanit Bouanil, Pipop Saikaew, Chantida Pawaputanon Na Mahasarakham

**Affiliations:** 1 Dental department, Phon Hospital, Khon Kaen, Thailand; 2 Department of Restorative Dentistry, Faculty of Dentistry, Khon Kaen University, Khon Kaen, Thailand; 3 Research Unit, Faculty of Dentistry, Khon Kaen University, Khon Kaen, Thailand; 4 Department of Operative Dentistry and Endodontics, Faculty of Dentistry, Mahidol University, Bangkok, Thailand

**Keywords:** Health care, Materials science

## Abstract

This study investigated the influence of silver diamine fluoride (SDF) on the shear bond strength (SBS) to artificial carious dentin and GIC restorations with various SDF application protocols. Artificial caries were prepared on human dentin discs using bacteria model. These samples were randomly allocated to five groups (n = 10/group) according to the following treatment: (1) control group (CD): no treatment (2) CSR: dentin conditioner, SDF, and rinsing (3) CS: dentin conditioner and SDF (4) SRC: SDF, rinsing and dentin conditioner, and (5) SC: SDF and dentin conditioner. The treated-dentin surface was bonded with GIC and subjected to SBS test. Mean SBS was analyzed using one-way ANOVA. Surface morphology and elemental contents after surface treatment were examined (n = 3/group) by scanning electron microscopy with energy-dispersive X-ray spectroscopy (SEM/EDX). There was no significant difference in the mean SBS among CD (2.45 ± 0.99 MPa), CSR (1.76 ± 0.65 MPa), and SRC (2.64 ± 0.95 MPa). Meanwhile, the mean SBS of CS (0.35 ± 0.21 MPa) was significantly lower than the control and SRC group. SEM/EDX demonstrated deeper silver penetration in CSR and CS groups when compared to SRC and SC groups. SDF-modified GIC restorations resulted in significantly lower bond strength in CS and SC groups. The findings suggested treating the carious dentin surface with CSR and SRC protocol. SDF-treated carious dentin should be rinsed off prior to restore with GIC.

## Introduction

Deep carious lesions can be challenging to restore in clinical practice. Traditional guidelines advocate nonselective caries removal to control cariogenic activity and provide well-mineralized dentin to achieve successful and long-lasting restorations^1^. This method can easily lead to accidental pulpal exposure, which is considered a highly unfavorable prognostic factor^1,2^. Recently, so-called “selective carious tissue removal” was proposed. With this technique, peripheral caries are completely removed, whereas the firm and leathery carious dentin over the pulpal surface is left. Therefore, the chance of pulp exposure is minimized^2^. However, leaving carious dentin may compromise restoration longevity, mainly because of mechanical and adhesive reasons^3–6^. Ricucci et al.^7^ demonstrated that the residual bacteria underneath the restoration provoked an inflammatory response with an absence of clinical symptoms during the entire observation period. Therefore, there is a risk of clinical failure due to reinfection or pulpitis when viable bacteria are present in dentin^7,8^.

Silver diamine fluoride (SDF) is a topical fluoride used to clinically prevent and arrest dental caries in young children^9–12^ and older adults^13–15^. The combination of silver and fluoride has a synergistic effect that arrests active dentin caries^16^. Regarding the antibacterial effect, silver ions react with the thiol groups of amino and nucleic acids, disrupting bacterial metabolic and reproductive pathways and inducing cell death^17,18^. Furthermore, the reactivation of SDF (zombie effect) is also observed when dead silver-containing bacterial cells make contact with and kill living bacterial cells. This reservoir effect helps explain why silver deposited on bacteria and dentin proteins within a cavity has sustained antimicrobial effects^19^. Meanwhile, fluoride ions promote the remineralization of dental tissues by forming fluorapatite and fluorohydroxyapatite on the tooth surface^16^. Due to these benefits, it has been suggested to apply SDF before restoration to prevent secondary caries lesions and improve the biomechanical and biochemical properties of carious dentin^20,21^.

SDF has been recommended for application to residual carious dentin prior to permanent restoration with glass-ionomer cement (GIC) in a silver modified atraumatic restorative treatment (SMART) procedure^22–24^. This technique is often used in transitional or definitive restorations for high-risk caries or less cooperative patients, including providing care outside the dental office^25^. However, the results from previous studies are inconsistent^26–32^. This could be due to the lack of consensus regarding the protocol for SDF application, (39) which might be related to the success of the bonding performance. To date, no studies have directly compared the effectiveness of surface conditioners and rinsing steps immediately after SDF treatment to the bonding ability of GICs on carious dentin. Therefore, the aim of this study was to investigate the influence of SDF on the shear bond strength (SBS) of artificial carious dentin and GIC restorations with various SDF application protocols. The null hypothesis in the current study was that the application protocol does not affect the SBS of GIC and the amount of silver remnant.

## Materials and methods

### Specimen preparation

The protocol of this study was reviewed by the Ethics Committee for Human Research of the local university (HE622155). Sixty-five extracted human third molars were used in this study. All teeth selected for the experiment exhibited mature features of an intact crown and a root without a crack line on the surface. If either cavitated lesions or existing restoration was detected, the tooth was excluded from the study. All teeth were curetted to remove gingival tissues and calcium remnants and subsequently rinsed with distilled water prior to storage in 0.1% thymol solution at room temperature and used within 6 months.

The occlusal dentin disc, approximately 2 mm thick, was prepared perpendicularly to the long axis of teeth with a model trimmer to expose the flat dentin surface at a mid-coronal level. The first cut on the crown was created 2 mm from the cementoenamel junction, and the second cut was performed 2 mm from the first cut and parallel to it. Then, all dentin discs were disinfected under UV light for 1 h on each side before the experiment.

### Cariogenic biofilm challenge

The microorganisms used for the cariogenic biofilm challenge were *Streptococcus mutans* (ATCC 25,175) and *Lactobacillus acidophilus* (ATCC 4356). The bacteria were cultivated on brain heart infusion (BHI) agar plates and de Man Rogosa and Sharpe (MRS) agar plates until isolated colonies were visible (5% CO_2_ concentration, 37 °C for 48 h). Next, the grown colonies were collected and separately resuspended in tubes containing MRS broth and incubated for 24 h. At the end of this culture period, the concentration of each bacteria was adjusted to 10^7^ CFU/mL using a spectrophotometer wavelength of 600 nm (the optical density was 0.5). Subsequently, each bacterial suspension was equally mixed (1:1 ratio of mixture) in 5% sucrose solution and incubated for at least 1 h or until the pH was 5–6. Finally, 1 mL of bacterial suspension was incubated with each dentin disc in a 12-well plate. The plates were incubated for 3 days, and the medium was refreshed daily to induce the formation of carious lesions.

### Experimental treatment

**Figure 1:**
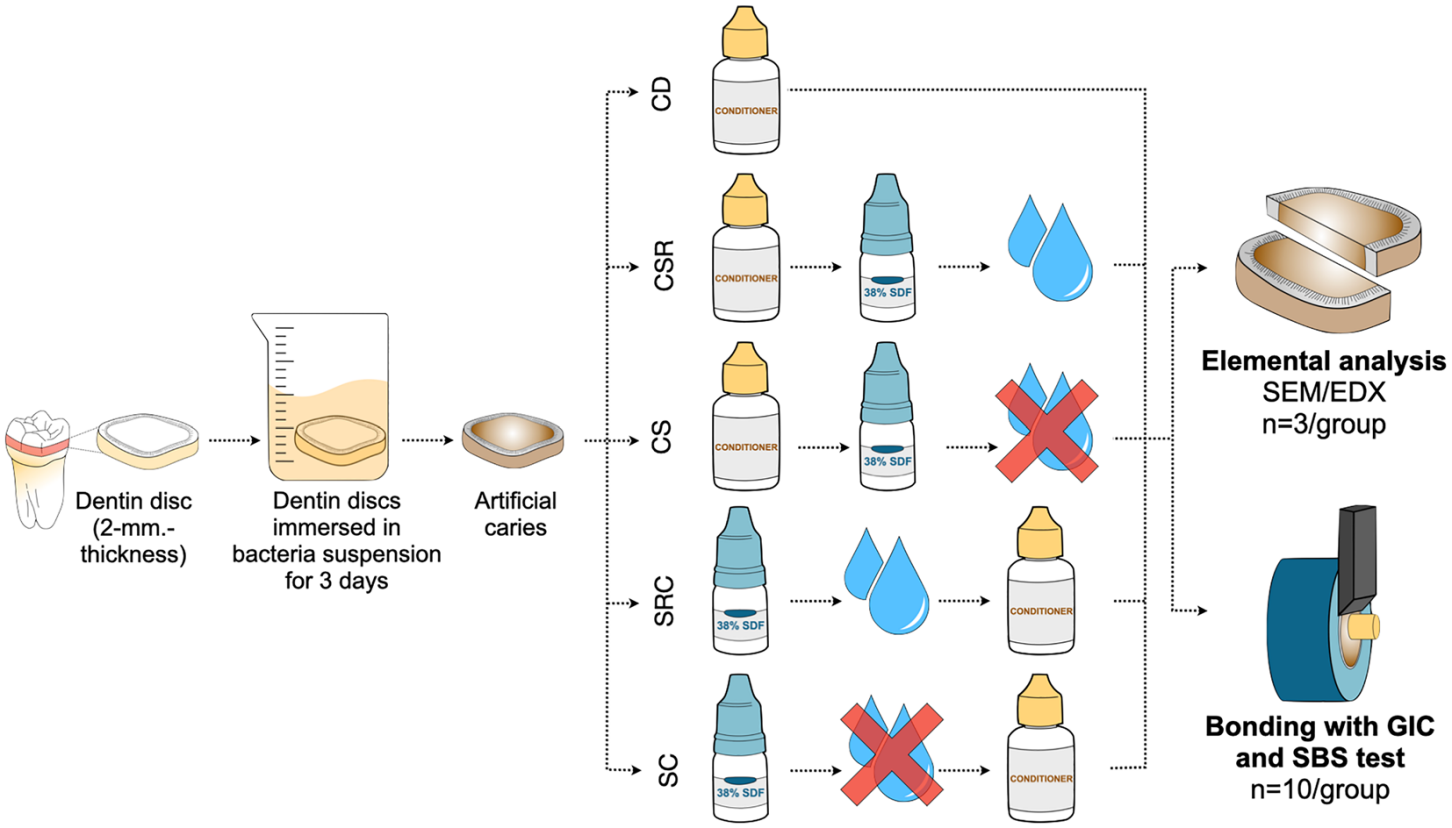
A schematic illustration representing the procedures used in the current study. *SDF* 38% silver diamine fluoride; *GIC* conventional glass ionomer cement; conditioner conditioner, 10% polyalkenoic acid; *CD* Carious dentin; *CSR* dentin conditioner + SDF + rinsing with water; *CS* dentin conditioner + SDF + no rinsing; *SRC* SDF + rinsing with water + dentin conditioner; *SC* SDF + dentin conditioner.

A schematic illustration of the study design is shown in Fig. 1. Sixty-five carious dentin discs were divided into five subgroups, and the dentin surfaces were treated with the following protocols, as shown in Table 1.

**Table 1:** Application procedure of SDF treatment.

Group	Application procedure
CD	The carious dentin was used as a baseline. This group was cleaned with dentin conditioner for 20 s, then rinsed and dried
CSR	The carious dentin was treated with dentin conditioner for 20 s, followed by rinsing and drying. Then, 38% SDF was applied to the carious dentin by scrubbing for 1 min, leaving for 3 min, then rinsing thoroughly, and gently drying
CS	The carious dentin was treated with dentin conditioner for 20 s, followed by rinsing and drying. Then, 38% SDF was applied to the carious dentin by scrubbing for 1 min, leaving for 3 min (no rinsing), and gently drying
SRC	The carious dentin was treated with 38% SDF by scrubbing for 1 min, leaving for 3 min, rinsing thoroughly, and gently drying. Then, dentin conditioner was applied to the carious dentin for 20 s, then rinsed and dried
SC	The carious dentin was treated with 38% SDF by scrubbing for 1 min, leaving for 3 min (no rinsing), and gently drying. Then, dentin conditioner was applied to the carious dentin for 20 s, then rinsed and dried

### Assessment of shear bond strength (SBS) and fracture mode

Immediately after dentin discs were treated with the protocol, the biofilm on the carious dentin surface was removed by ultrasonication before GIC restoration. After that, a plastic tube 4 mm in diameter and 2 mm in height was placed on the treated surface. GICs were mixed using a rotational capsule mixing unit (Fuji IX GP Extra Capsule, GC America Inc.) for 10 s, and then the mixture was injected into a plastic tube to form a cylindrical button. The GIC was compressed with a mylar strip to ensure good adaptation. After 5 min of setting, the tube was removed, and the GIC-dentin specimens were stored at 100% relative humidity and incubated at 37 °C for 24 h to ensure GIC maturation before the bond strength test.

The SBS test (n = 10/group) was performed with a universal testing machine with a knife-edge loading head (Universal testing machine; LR30K, Lloyd instruments, UK). A shear force was applied perpendicularly to the GIC–dentin interface with loading at a fixed rate of 1 mm/minute. The maximum load used to debond GICs was recorded in newtons. The bond strength was expressed in megapascals (MPa) by dividing the load at failure by the bonded surface area in square millimeters.

The fracture surface was examined under an optical microscope at 20 × magnification. The failure modes were categorized into three types:

- Type 1: adhesive failure between dentin and GICs and dentin surfaces.
- Type 2: cohesive failure in GICs or dentin.
- Type 3: mixed failure; partially adhesive failure and partially cohesive failure.

### Assessment of surface morphology and elemental analysis

Fifteen carious dentin discs were prepared as previously described. After treatment according to the 5 experimental groups (n = 3), all specimens were stored at 100% relative humidity and incubated at 37 °C for 24 h. Then, the specimens were carefully fractured (long axis) by a scalpel blade and a hammer and dried at room temperature for 24 h.

One half of the fractured specimen was examined through **elemental mapping analysis** to detect the presence and distribution of silver, fluoride, phosphorous, and calcium ions of the precipitate on the surface of the specimens (n = 3/group) via energy–dispersive X–ray spectrometry (EDX) under scanning electron microscopy (SEM) at a magnification of 2500x (SEM, SU3800, Hitachi Ltd., Tokyo, Japan).

The exposed cross-section surface of the other half of the fractured specimen was examined using **elemental line analysis** to determine the deposition of silver ions and other ions at the targeted line via EDX under SEM. The targeted line, which was 250 μm in length from the surface of the specimen toward the pulp, was randomly selected.

### Statistical analysis

Data were analyzed using SPSS Premium V.28 for Mac OS (IBM, Armonk, NY, USA) for statistical analysis. The SBS data were reported in the study as means and SDs. The normality of data was assessed using the Shapiro–Wilk test. For normally distributed results, one-way ANOVA followed by the Dunnett T3 test post hoc multiple-comparison test was employed to analyze the SBS data. In addition, the failure mode was descriptively reported as a percentage. For all the analyses, the significance level was set at *P–value* < 0.05.

The post hoc power analysis was performed using G*Power version 3.1.9.6 (University of Dusseldorf, Dusseldorf, Germany). The effect size of the SBS experiment was calculated from the results obtained in the current study, which demonstrated that the sample size used in each test exhibited a power > 0.95 at alpha = 0.05.

Finally, the SEM/EDX images of silver ions and other deposition data were used for descriptive analysis.

### Ethics approval

All procedures performed in this study were in accordance with the principles of the Declaration of Helsinki. The ethics committee of Khon Kaen University approved this study with the code HE622115.

### Informed consent

All specimens could not identify the participants, and their information was anonymous. For the above reasons, the need for informed consent has been waived by Khon Kaen University Ethics Committee for Human Research.

## Results

### Assessment of shear bond strength (SBS) and fracture mode

**Figure 2:**
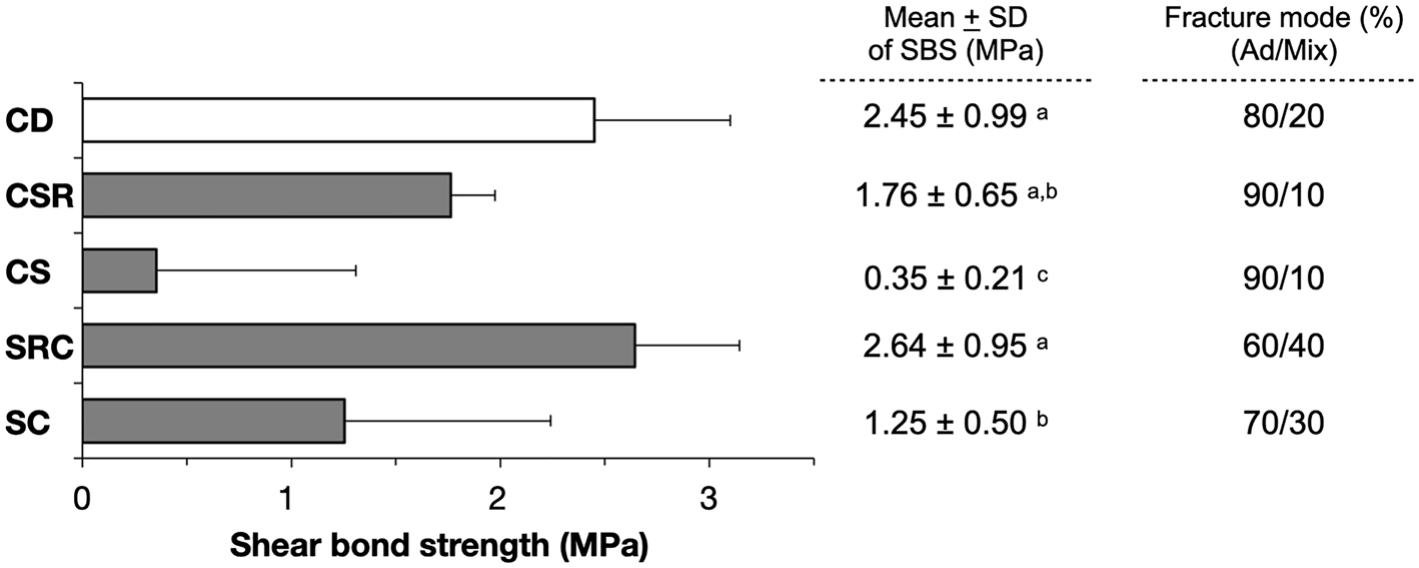
Mean of SBS (MPa) and percentage of fracture mode of GIC to carious dentin after SDF application with various surface treatment methods. Different letters indicate statistically significant differences at *P* < 0.05 with Dunnett T3 post hoc test. *SBS* shear bond strength; *CD* Carious dentin; *CSR* dentin conditioner + SDF + rinsing with water; *CS* dentin conditioner + SDF + no rinsing; *SRC* SDF + rinsing with water + dentin conditioner; *SC* SDF + dentin conditioner; *Ad* adhesive failure, and *Mix* mix failure. All data analyzed during this study are included in the Supplementary Information.

No pretest failure was observed in this study. The means and standard deviations of SBS are presented in Fig. 2. The highest SBS (2.64 ± 0.95 MPa) was observed in the SRC group, whereas the lowest SBS (0.35 ± 0.21 MPa) was observed in the CS group. Only the CSR and SRC groups demonstrated no significant difference compared to the CD group (CSR; *P* = 0.521, SRC; *P* > 0.05, respectively).

Adhesive failure was the most prevalent type of failure in all groups regardless of the surface treatment (Fig. 2 and Supplementary Information). Notably, an increase in mixed failure was observed in the groups treated with conditioner after SDF treatment (SRC and SC).

### Assessment of surface morphology and elemental analysis

**Figure 3:**
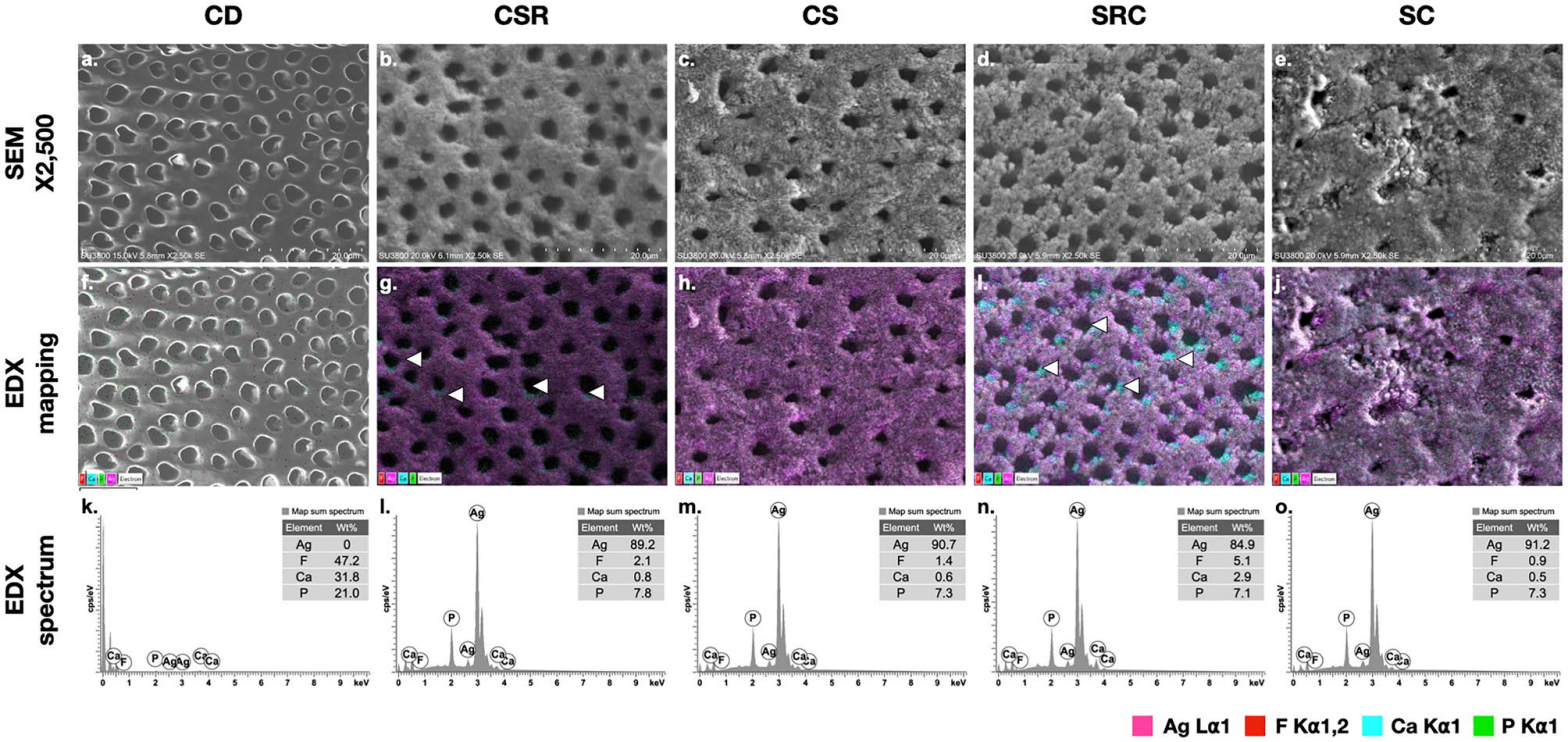
SEM at 2500X magnification (**a**–**e**), EDX elemental mapping (**f**–**j**) and EDX spectra of carious dentin (**k**–**o**), after SDF application with various surface treatment methods. *CD* Carious dentin; *CSR* dentin conditioner + SDF + rinsing with water; *CS* dentin conditioner + SDF + no rinsing; *SRC* SDF + rinsing with water + dentin conditioner; *SC* SDF + dentin conditioner; *Ag* silver (pink); *F* fluoride (red); *Ca* calcium (blue); *P* phosphorus (green). White arrows indicate the exposed calcium ion.

Figure 3 shows representative images of the SEM/EDX analysis. In the CD group, the smear layer and smear plug on the surface were absent (Fig. 3a). In addition, all dentinal tubules were opened with demineralized peritubular dentin. The CD group showed a decrease in calcium and phosphate compared to healthy dentin (Supplementary Information). Notably, silver deposition was not detected in the CD group (Fig. 3f and k).

For the experimental groups, the entire surfaces were covered with a silver–enriched layer (Fig. 3g–j), especially the CS (Fig. 3h) and SC (Fig. 3j) groups, in which other elements underneath the silver layer were barely detected. Moreover, the CSR and SRC groups exhibited a loose silver layer, leading to the detection of the colocalization of silver and calcium in some areas in the CSR group (Fig. 3g) and the robust detection of the same in the SRC group (Fig. 3i). Notably, all dentinal tubules were opened in the CSR and SRC groups (Fig. 3b and d, respectively).

**Figure 4:**
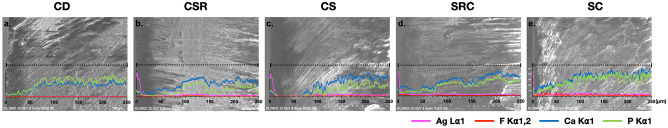
SEM at 500X magnification (**a**–**e**) and EDX line–scan representing element profile (Ag, F, Ca, and P element) along the path (line in **a**–**e**). *CD* Carious dentin; *CSR* dentin conditioner + SDF + rinsing with water; *CS* dentin conditioner + SDF + no rinsing; *SRC* SDF + rinsing with water + dentin conditioner; *SC* SDF + dentin conditioner; *Ag* silver.

The SEM/EDX elemental line analysis is shown in Fig. 4a–e. All groups revealed a decrease in calcium and phosphate until approximately 80 μm depth compared to sound dentin, which presented a high intensity of calcium and phosphate along the cross-sectional surface (Supplementary Information). In addition, a silver-enriched layer of approximately 20 μm was found on the carious dentin surface in the CSR and CS groups (Fig. 4b and c, respectively), which was thicker than that in the SRC and SC groups at approximately 10 μm thickness (Fig. 4d and e, respectively).

## Discussion

This study investigated the influence of various SDF treatment protocols on artificial carious dentin induced by biofilms before restoration with GIC. According to the results of this study, normal dentin presented higher SBS than carious dentin (Supplementary Information). The SBS of GIC of the carious dentin (CD) group was not significantly different from those of the SRC and CSR groups (*P* > 0.05). However, significantly lower SBS was observed in the CS and SC groups (*P* < 0.05). Therefore, the null hypothesis was rejected.

It was reported that a silver phosphate was formed on the SDF-treated dentin surface after SDF application^33^, and free silver particles extended into the dentinal tubules, totally or partially obstructing them^34^. These mechanisms might affect both the mechanical interlocking and chemical bonding of the GIC to SDF-modified dentin. Therefore, many publications have recommended eliminating excess silver precipitation to minimize the negative effect of SDF on dentin^29,30,32,35–37^. However, previous studies have reported contradictory outcomes regarding the nonstandard protocol for SDF application. Some studies demonstrated that SDF had no effect on the bond strength of GIC to dentin^26,27,29,30^, while others reported that SDF reduced the bond strength^32,38^. These inconsistent results might be due to the different surface treatment protocols used in the previous studies.

This study reveals that the bond strength of GIC to artificial carious dentin was affected by SDF and the different surface treatment procedures. The SDF application without removing excess silver precipitate through a rinsing step, as in the CS and SC groups, led to a significantly lower bond strength compared to that in the untreated carious dentin surface (CD group). This finding is in agreement with previous studies^32,38^. This could be due to residual silver that hinders the adhesion between GIC and underlying dentin. This is supported by our EDX analysis demonstrating the dense silver-enriched layer that covered the dentin in the CS and SC groups (Fig. 3c and e, respectively). Such a layer can alter the surface energy and the degree of wetness on the dentin surface^39^, impairing the binding of GIC to the underlying dentin. Moreover, this layer probably also prevents intimate contact between GIC and dentin. Therefore, there is less opportunity for bonding between the carboxylate groups in the GIC and the calcium ions from the hydroxyapatite^40^. Finally, the SDF-treated surfaces without rinsing treatment may present excessive alkalinity due to the high pH of SDF^36^. Such pH can impede the acid–base reaction of GIC and adversely affect the tooth-GIC interface^40^.

Regarding the CSR and SRC groups, no statistical significance compared to the untreated carious dentin surface was detected. This is supported by previous studies demonstrating no effect of SDF on the bond strength when a rinsing step was applied before GIC restoration^26,27,29,30^. The adverse effect on the bond strength of GIC seems to be eliminated by rinsing SDF off before GIC restoration. However, the sequence of dentin conditioner application (before or after SDF application) did not affect the bond strength of GIC to SDF-treated carious dentin.

When the rinsing step was applied after SDF application, the excess free silver ions might be removed by the rinsing water from the superficial surface. Therefore, a looser silver-enriched layer and obvious open dentinal tubules were observed in the CSR and SRC groups (Fig. 3g and i, respectively). This technique may be practical for modifying SDF-treated surfaces, allowing freshly placed GIC to penetrate the underlying dentin, leading to a proper micromechanical interlocking and chemical interaction^39^. Additionally, EDX elemental mapping revealed the presence of calcium ions on the SDF-treated surfaces in the CSR (Fig. 4b) and SRC groups (Fig. 4d). This result might facilitate the chance of developing the chemical bond between calcium ions from the hydroxyapatite and carboxylate functional groups from the GIC^41^, which likely improves the SBS in the SRC and CSR groups. On the other hand, no calcium ions were detected in the SC and CS groups, which is consistent with previous studies^36,42,43^. Therefore, a rinsing step after SDF application is strongly recommended to minimize free silver precipitation and reduce the alkalinity of the SDF-treated surface. This protocol may enhance the bond strength for long-lasting restoration, including maintaining the SDF therapeutic effect due to the residual silver precipitates on the SDF-treated surface.

When SDF is applied to the tooth structure, silver ions are released from the diamine silver ion complex, leading to the penetration and deposition of silver particles into the tooth structure^44^. This study showed that the silver-enriched layer thickness on artificial carious dentin was up to 20 µm using EDX line scanning analysis. However, a difference in silver-enriched layer thickness among groups was observed, which might depend on the order of PAA application. As demonstrated in Fig. 3a (CD group), PAA is a mild acid^45^ that can remove the smear layer and facilitate partial demineralization. Therefore, if SDF is applied on the PAA-treated carious dentin surface, the zone of exposed collagen is subsequently infiltrated with silver particles^46,47^ to form a thicker silver-enriched layer, as found in the CS and CSR groups. This finding indicated that dentin surface conditioning with PAA before SDF application could exert a positive effect. This probably promotes silver ion deposition deep into artificial carious dentin, resulting in a thorough silver-based antimicrobial effect^48,49^. Furthermore, the remaining silver ions probably continue to react with hydroxyapatite, forming silver phosphate (Ag_3_PO_4_). This compound might transform into other compounds and release phosphate ions to initiate apatite formation, leading to the remineralization of demineralized dentin^50^. Moreover, the presence of stable silver precipitates can introduce antimicrobial and anticariogenic properties and, thus, may be very useful for improving the resistance of restorations to secondary caries. Additionally, various forms of particle silver aggregates may improve the mechanical properties of this altered carious dentin substrate. However, the mechanism of particle formation might need further investigation.

The clinical implication of this finding is that if SDF is used to treat residual caries prior to GIC restoration, the bond strength of GIC on the carious dentin might be impaired. However, the beneficial effect of rinsing SDF off was demonstrated, which seems to eliminate this adverse effect. Therefore, for SDF-modified GIC restorations, it is suggested to apply SDF and rinse it off before PAA application, as in the SCR group. Applying PAA before SDF, as in the CSR group, is also favorable since it facilitates more effective silver ion absorption into the collagen network. However, the effect of SDF precipitated on dentin in long-term studies or randomized clinical trials might be necessary.

## Conclusion

SDF-modified GIC restoration resulted in significantly lower bond strength in the CS and SC groups. The findings supported the treatment of carious dentin surface with CSR and SRC protocols.

### Supplementary Information

Supplementary Information 1.

Supplementary Information 2.
