# Supplementary material for: Bonding performance of glass ionomer cement to carious dentin treated with different surface treatment protocols using silver diamine fluoride
Source: Sci Rep. 2023 Aug 30;13:14233. doi: 10.1038/s41598-023-41511-9 (PMC10468524; doi:10.1038/s41598-023-41511-9)
Supplement: Supplementary file 1 — Supplementary Information 1. [file 41598_2023_41511_MOESM1_ESM.docx]

**Supplementary information I**


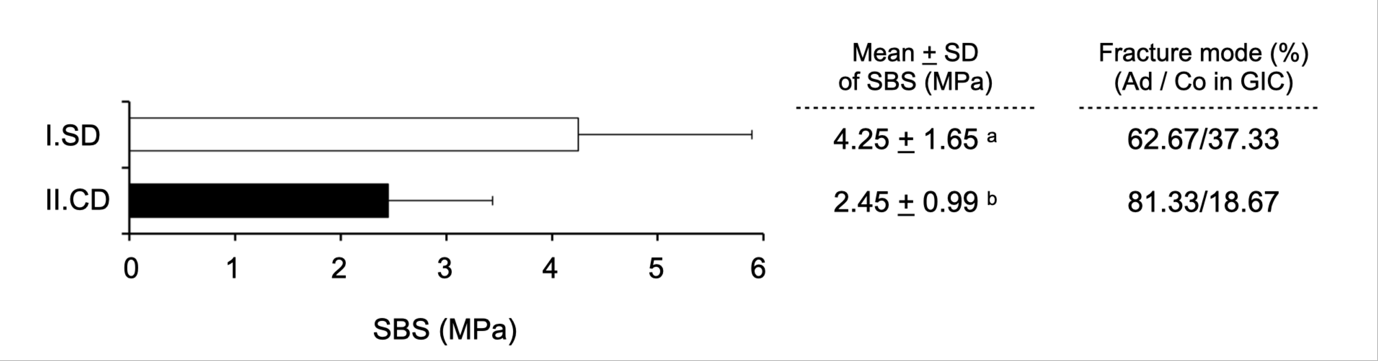


Fig. 1 Mean of SBS (MPa) and percentage of fracture mode of GIC to sound dentin (SD) and carious dentin (CD). Different letters indicate statistically significant differences at *P* < 0.05 with Dunnett T3 post hoc test.


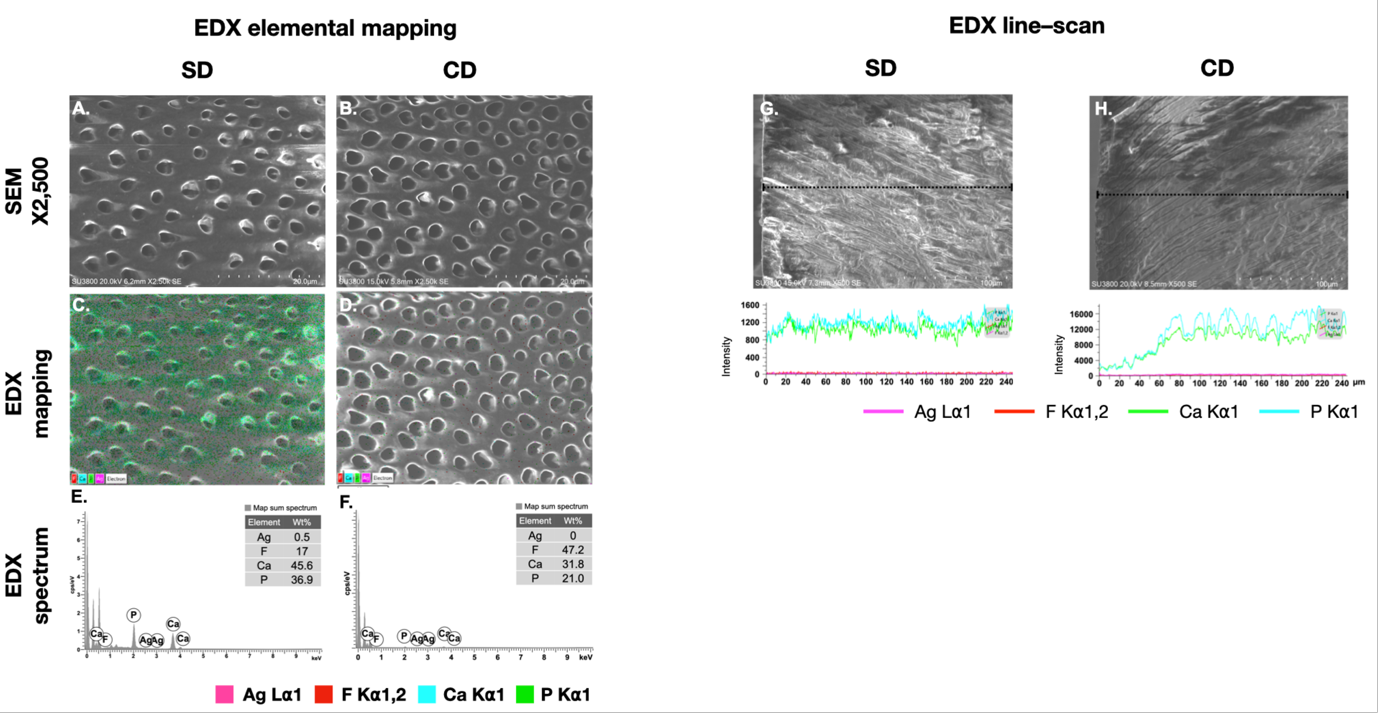


Fig. 2 A-F; SEM at 2,500X magnification, EDX elemental mapping and EDX spectra of sound dentin (SD) and carious dentin (CD). G-H; SEM at 500X magnification) and EDX line–scan representing element profile (Ag, F, Ca, and P element) along the path.


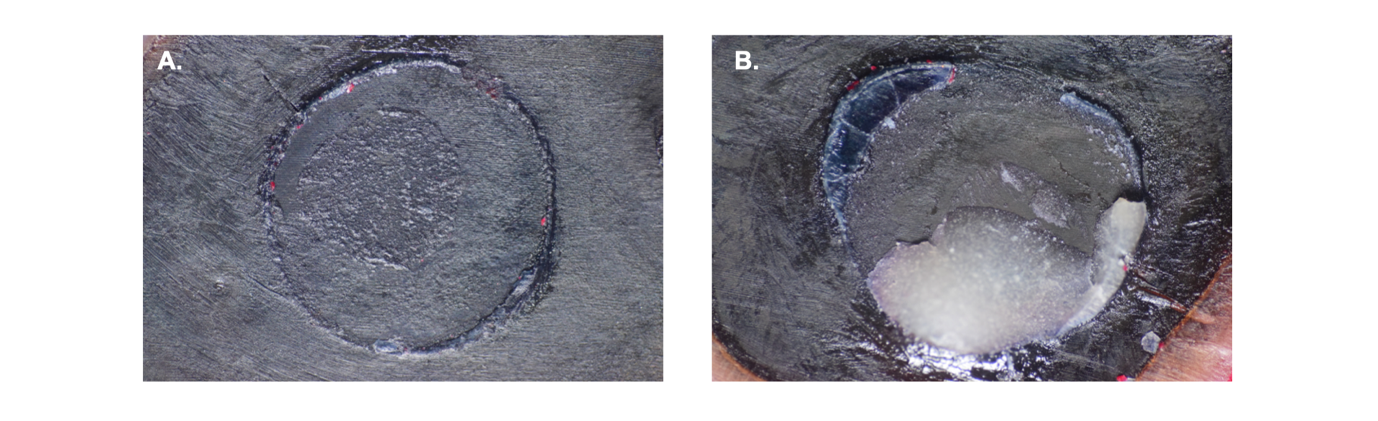


Fig. 3 A-B; An optical microscope at 20x magnification, photographs of fracture surfaces for each failure mode (A) adhesive failure, 75% to 100% failure occurred at interface of GIC dentin bond (B) Mixed failure, mixed with adhesive failure at the GIC/dentin interface and cohesive failure in GIC

Table 1 Shear Bond Strength (MPa), Mean and standard deviation of SBS

|  | CD | CSR | CS | SRC | SC | Sound |
| --- | --- | --- | --- | --- | --- | --- |
| 1 | 2.13 | 1.25 | 0.07 | 3.84 | 0.44 | 4.11 |
| 2 | 2.86 | 2.86 | 0.49 | 1.34 | 1.30 | 6.47 |
| 3 | 1.92 | 2.08 | 0.19 | 2.54 | 0.68 | 5.13 |
| 4 | 1.49 | 1.42 | 0.11 | 2.90 | 1.96 | 6.37 |
| 5 | 2.58 | 1.16 | 0.59 | 1.50 | 1.55 | 4.78 |
| 6 | 2.17 | 1.66 | 0.72 | 3.98 | 1.30 | 3.16 |
| 7 | 2.85 | 1.20 | 0.39 | 2.42 | 1.04 | 5.37 |
| 8 | 3.34 | 2.78 | 0.32 | 3.71 | 1.13 | 1.65 |
| 9 | 0.83 | 1.20 | 0.42 | 2.33 | 1.10 | 2.84 |
| 10 | 4.34 | 2.01 | 0.21 | 1.87 | 2.02 | 2.61 |
| Mean | 2.45 | 1.76 | 0.35 | 2.64 | 1.25 | 4.25 |
| SD | 0.99 | 0.65 | 0.21 | 0.95 | 0.50 | 1.65 |
