# Supplementary material for: Bonding performance of glass ionomer cement to carious dentin treated with different surface treatment protocols using silver diamine fluoride
Source: Sci Rep. 2023 Aug 30;13:14233. doi: 10.1038/s41598-023-41511-9 (PMC10468524; doi:10.1038/s41598-023-41511-9)
Supplement: Supplementary file 2 — Supplementary Information 2. [file 41598_2023_41511_MOESM2_ESM.docx]

**Supplementary information II**

1. Assessment of biofilm formation after cariogenic biofilm challenge by SEM

The formation of a biofilm monolayer was observed on the first day. Subsequently, on day 5 of incubation, a matured biofilm with multiple layers was observed.


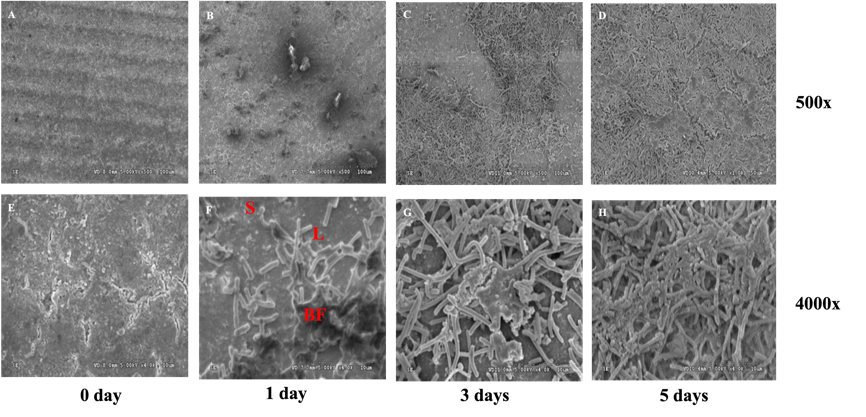


Fig 1 Scanning electron microscope (SEM) image of biofilm grown on dentin discs at 500X and 4,000X magnification.

S, *Streptococcus mutans;* L, *Lactobacillus acidophilus;* BF, *Biofilm formation*

1. Assessment of lesion depth and mineral content loss after cariogenic biofilm challenge by SEM/EDX elemental line analysis

Artificial carious dentin (CD) revealed a decrease in calcium and phosphate until approximately 80 μm depth, whereas sound dentin (SD) exhibited high intensity of calcium and phosphate across its cross-sectional surface.


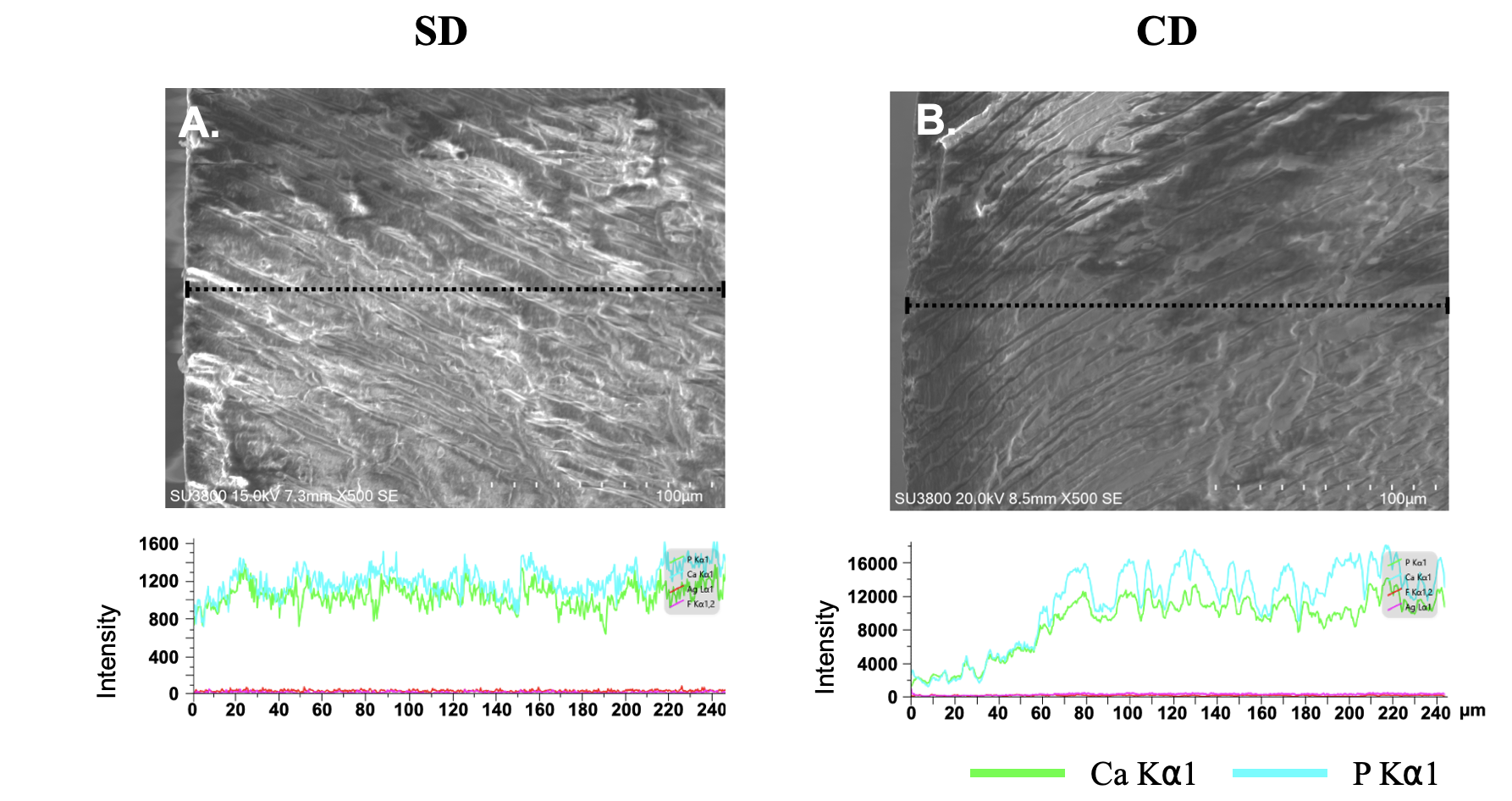


Fig 2 SEM at 500X magnification (A – B) and EDX line–scan representing element profile (Ca, and P element) along the path of sound dentin (SD) and carious dentin (CD).

1. Assessment of dentin hardness after cariogenic biofilm challenge by Vicker microhardness

Artificial carious dentin presented a decrease in Vicker hardness compared to sound dentin, as shown in Table 1.

| Table 1 Surface hardness in the sound dentin and carious dentin | |
| --- | --- |
| Group | Surface hardness  (mean + SD) VH |
| SD | 49.65 + 5.89 |
| CD | 2.63 + 0.59 |
| SD, sound dentin; CD, carious dentin; VH, Vicker’s hardness  Test condition for SD: load 50 g, 10 s; for CD: 10 g, 10 s  N = 5/group | |
